# Development of the sterile insect technique as a control strategy for the Asian citrus psyllid: determining the number of sterile individuals needed to suppress the growth of wild populations

**DOI:** 10.1093/jee/toag116

**Published:** 2026-04-27

**Authors:** Jedeliza B Ferrater, Gregory S Simmons, Matthew P Daugherty, Sandy Priego Solis, Mya Rodriguez, Sen Miao, Paul F Rugman-Jones

**Affiliations:** Department of Entomology, University of California Riverside, Riverside, CA, USA; US Department of Agriculture, APHIS, PPQ-S&T California Station, Salinas, CA, USA; Department of Entomology, University of California Riverside, Riverside, CA, USA; Department of Entomology, University of California Riverside, Riverside, CA, USA; Department of Entomology, University of California Riverside, Riverside, CA, USA; Department of Entomology, University of California Riverside, Riverside, CA, USA; Department of Entomology, University of California Riverside, Riverside, CA, USA

**Keywords:** *Diaphorina citri*, citrus greening, vector control, X-ray irradiation, IPM

## Abstract

Controlling the Asian citrus psyllid (ACP), *Diaphorina citri*, is key to preventing spread of huanglongbing, a disease that has decimated citrus industries worldwide. One promising control method is the Sterile Insect Technique (SIT). This involves rearing large numbers of a target pest, sterilizing them using radiation, and releasing them into the field to compete with wild conspecifics for mating, ultimately suppressing population growth. The success of SIT is largely influenced by 3 factors: (i) ensuring released insects are sufficiently sterile such that mating between wild and sterile individuals yields no (or very few) offspring; (ii) ensuring the ratio of sterile to wild insects released (the overflooding ratio [OFR]) is sufficient to minimize the occurrence of mating between 2 wild individuals; and (iii) ensuring the released insects are competitive with respect to mating performance, dispersal, and longevity. Previously, we tentatively identified that a 320 Gy dose of X-ray radiation was required to sterilize ACP. Herein we investigate the impact of OFR on the growth of caged populations. Laboratory-cage experiments suggested that following sterilization with 240 or 320 Gy, a single release of ACP at OFRs of 10:1 and 15:1, could effectively suppress subsequent growth of ACP populations. To validate this finding under more natural conditions, we repeated the experiments using mature citrus trees held within field-cages. We found that a sterilizing dose of 320 Gy of X-ray radiation had the greatest impact in the field-cages, with an increase in the OFR from 10:1 to 15:1 showing only a marginal additional benefit.

## Introduction

The Asian Citrus Psyllid (ACP), *Diaphorina citri* Kuwayama (Hemiptera: Psyllidae), continues to present a significant threat to citrus production globally, including vast regions of the United States. ACP vectors the bacterium *Candidatus Liberibacter asiaticus* (*C*Las), which is associated with the highly destructive citrus disease huanglongbing (HLB), also known as citrus greening (Halbert and Manjunath 2004, Bové 2006, Li et al. 2021). Infection of citrus trees with HLB leads to declining tree vigor, diminished yields, reduced fruit quality, and ultimately death of the entire tree, typically within 2 to 4 years (Tipu et al. 2021, Pulici et al. 2022, Shahzad et al. 2023). Among the citrus-producing regions in the U.S., Florida has been hit particularly hard by HLB, the result being a more than 90% drop in overall citrus production over the past 20 years, which has fundamentally altered the agricultural landscape (Shah 2024). In contrast, the incidence of HLB in California remains relatively low, and the major area of production, the San Joaquin Valley, continues to be free from HLB.

There is currently no cure for HLB, and all types of citrus and cultivars are affected (Grafton-Cardwell et al. 2013, da Graca et al. 2016, Li et al. 2021), resulting in efforts being directed toward control of the vector, to limit disease incidence. Different approaches are used to manage ACP depending on its establishment level. In Florida, where ACP is firmly established, growers employ multiple suppression strategies including chemical control, physical barriers (exclusion mesh and protective covers), and classical biological control to reduce ACP populations (Diepenbrock et al. 2022). California has adopted a more preventative approach since ACP is not yet widespread in the state, aside from established populations in southern California (UC ANR 2025). California’s strategy employs a multi-pronged approach to combat HLB: rapidly eliminating diseased trees via improved surveillance and mandatory removal protocols while partnering with scientists on early detection technologies; restricting psyllid movement through stringent enforcement of regional quarantines to prevent moving host plants, leaves, or nursery stock; suppressing psyllid populations using coordinated area-wide treatments, releases of biocontrol agents, and removal of abandoned host plants alongside continuous assessment of urban treatment methods; modernizing data systems to improve analytics, sharing, and digital capabilities; and mobilizing community support through targeted outreach that engages homeowners, industry stakeholders, and local government partners (California Department of Food and Agriculture [CDFA] 2025).

Treatments for ACP are strategically timed to coincide with periods when citrus trees produce flush; the soft, young shoots that ACP favor for feeding and egg-laying (Grafton-Cardwell et al. 2013, Garcia-Figuera et al. 2022). However, in non-commercial citrus settings (typically homeowners’ backyard trees), pesticide application is deemed impractical due to concerns about the costs of treating such an extensive area, the risks of using chemicals in densely populated urban environments, and growing regulatory limitations on pesticide usage (California Department of Pesticide Regulation 2023). This situation prompted the implementation of a classical biological control program in California to help provide control in urban and peri-urban areas through the release of an imported parasitoid, *Tamarixia radiata* (Milosavljević et al. 2017, 2022).

To complement this existing classical biological control program, we are assessing the sterile insect technique (SIT) as an additional management option to limit the impacts of ACP and HLB in newly invaded areas. SIT is an environmentally friendly, pesticide-free method that has proven effective in the prevention and eradication of several important insect pests. In SIT, insects are mass-reared, exposed to a sterilizing dose of radiation, and released in the field. Following their release, the sterilized insects compete with wild conspecifics for mating opportunities, and any matings that involve these sterilized insects result in the production of no (or very few) offspring. If the chances of mating with a sterile partner are high enough, this can effectively suppress growth of the wild pest population (Knipling 1955). In comparison to other pest control methods, SIT is species-specific, does not release harmful substances or new genetic material into the environment, and creates no concern for non-target organisms (Hendrichs et al. 2002, Helsinki et al. 2009, Bourtis and Vreysen 2021). SIT has been successfully used to eradicate major pests such as the New World screwworm, *Cochliomyia hominivorax* (Krafsur et al. 1987, Lindquist et al. 1992), the tsetse fly, *Glossina austensi* (Vreysen et al. 2000, Klassen and Curtis 2005), and the pink bollworm, *Pectinophora gossypiella* (Simmons et al. 2021b, Tabashnik et al. 2021). The technique has also been employed to prevent the establishment of the Mediterranean fruit fly, *Ceratitis capitata* (Hendrichs et al. 2002, Enkerlin 2021, Plá et al. 2021) and for the area-wide suppression of both codling moth, *Cydia pomonella*, and false codling moth, *Thaumatotibia leucotreta* (Simmons et al. 2021a). With respect to ACP, SIT might be used to control new ACP infestations if they occur outside established quarantine zones. Alternatively, SIT may be employed as a preventive release strategy in buffer zones around infested areas to help limit their spread. As such, it could support ongoing management of ACP in areas where frequent pesticide applications are impractical, such as urban settings. Importantly, SIT is also compatible with existing integrated pest management (IPM) strategies for ACP, such as the release of *Tamarixia radiata* and the use of lower toxicity and lower residual insecticide treatments.

The first steps toward developing SIT as a control tool for ACP have already been undertaken. Efficient systems are already in place to rear large numbers of ACP to support the rearing of *T. radiata*, for biological control releases. Using curry leaf, *Bergera koenigii* L. (Rutaceae: Sapindales) (syn. *Murraya koenigii*), as a host plant, these systems can produce 5,000 to 7,000 ACP adults per square meter of greenhouse space within approximately 20 days (CDFA 2017). ACP production can be readily scaled up by increasing the number of *B. koenigii* plants and the available rearing space, as long as optimal temperature and humidity conditions are maintained. With this mass-rearing protocol using *B. koenigii* in place, the radiation biology of *D. citri* has also been investigated (Ferrater et al. 2024). Complete sterility in females could be achieved with a dose of 80 Gy of X-ray radiation, but males required a higher dose; a phenomenon that is not uncommon (Bakri et al. 2005). A dose of 320 Gy resulted in near 100% male sterility, with the mating of irradiated males with wild female ACP producing only a very small number of F1 progeny which themselves proved to be 100% sterile. This promising groundwork formed the basis for the current study, in which we further advance the development of SIT by determining the number of sterilized insects that would need to be released to affect population suppression; referred to as the overflooding ratio (OFR) [= number of sterilized ACP: wild ACP].

The primary goal of this study was to identify the most effective combination of radiation dose and OFR required to suppress the growth of a wild psyllid population using single SIT release. We initially tested 3 doses (240, 320, and 400 Gy) of X-ray radiation, along with 3 OFRs (5:1, 10:1, and 15:1), within laboratory cages housing small *B. koenigii* plants. Subsequently, we attempted to replicate the laboratory experiments by conducting releases on a bigger scale inside secure field cages housing mature lemon trees. In this second round of experiments, sterile ACP exposed to 240 or 320 Gy were released alongside wild ACP at release ratios of 10:1 or 15:1, allowing us to assess the effectiveness of sterile insects in locating and mating with wild ACP in a more complex and realistic (field-like) environment.

## Materials and Methods

### Host Plant Material

#### ACP Rearing and Laboratory Cage Experiments

Six-month-old *B. koenigii* plants were grown from seeds ­collected from the UCR Botanic Gardens. Seedlings were then transplanted into individual square plastic pots (8.3 L × 8.3 W × 8.9 cm H) containing UC Soil mix III and maintained in 4 greenhouses at Agriculture Operations, UCR as described in Ferrater et al. (2024). Plants are produced year-round under ambient temperature/humidity, and natural photoperiod, and used as required for rearing and in experimental assays. This relative of citrus is an excellent rearing host for our ACP colonies because it is not a host for HLB and the young plants are able to withstand continuous psyllid feeding (Skelley and Hoy 2004), thereby maximizing production in a limited space.

#### Field Cage Experiments

**Fig. 1.**
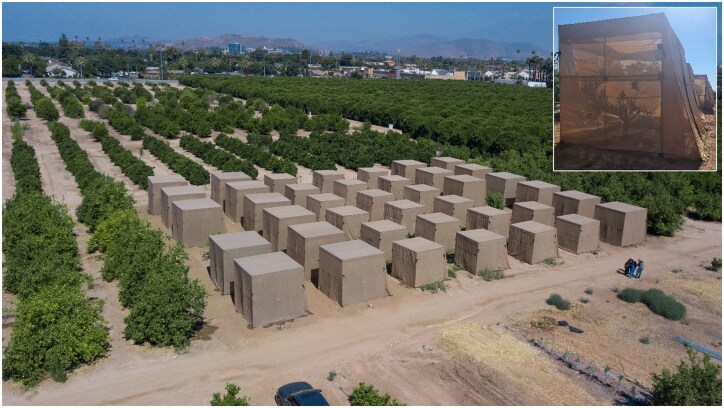
Caged lemon trees at Agricultural Operations, UCR, provide a more realistic, field-like environment to test the mating competitiveness and suppressive effect of irradiated ACP.

Trees used in the field cage experiments were mature lemon trees (*Citrus limon* L.) situated at the University of California, Riverside, Agricultural Operations facility. Trees were manually topped and hedged to a standardized height and canopy diameter of approximately 2.1 m, and skirted to remove any branches that hung lower than 60 cm from the ground. Individual trees were then enclosed within steel frame field cages measuring 3.05 × 3.05 × 3.05 m or 3.66 × 3.66 × 3.66 m (Fig. 1), and screened on the top and 4 sides by insect-proof (52 × 52 threads per inch = ∼0.3 mm mesh) polyethylene amber mesh (Lumite Inc., Alto, GA, USA). Cages were staked to the ground and a 50-cm wide polyethylene strip formed a skirted flap around the base of the cage that was weighed down with with ∼30 cm of soil, effectively sealing the cage to the ground, creating a physical barrier to insect movement that still allowed essential sunlight, air, and moisture to reach the tree. Human access to each cage was provided via a double-doored vestibule, with each door being closed by a sealed zipper before the next was opened. This setup minimized the risk of both ingress of unwanted insects and escape of experimental ACP. The trees were caged several months prior to the experimental releases, providing an extended period during which common residual pests, particularly citrus mealybug (*Planococcus citri*) and citrus leafminer (*Phyllocnistis citrella*), could be identified and removed. Over the course of 6 to 8 weeks, each tree underwent 3 inspections in which branches that displayed visible signs of pests were removed. All pruning debris was securely bagged and removed to prevent re-infestation. After the final inspection, the trees were sprayed with a commercial insect killing soap (49.52% potassium salts of fatty acids) on 3 occasions to eliminate any lingering arthropod pests, and a copper sulfate solution to inhibit the spread of sooty mold associated with mealybugs. To address Argentine ants (*Linepithema humile*) that are common in Southern California, we introduced commercial boric acid ant bait stations (5.4% sodium tetraborate decahydrate) to each cage. Finally, 10 to 14 days before our experimental releases, the “cleaned” trees received a final pruning, and were fertilized with a fish-based fertilizer (5-1-1) to promote general tree health and stimulate new vegative growth (increased flush) for ACP feeding and oviposition.

### Initiation of Experimental Psyllid Colonies

Adult *D. citri* were collected from a laboratory colony maintained at the University of California Riverside (UCR), Insectary and Quarantine Facility (I&Q). This colony, established in 2011 from field collections on citrus trees in southern California, is reared continuously on *B. koenigii*, and regularly screened to ensure its *C*Las-free status. The sex ratio of the colony is also monitored and, although it fluctuates slightly from generation to generation, it typically is close to 1:1 (data not shown). In preparation for each round of experiments, insect-rearing cages (47.5 × 47.5 × 47.5 cm; MegaView Science Co. Ltd., Taiwan) containing fifteen 6-month-old plants exhibiting active flush, were infested with approximately 200 psyllids (of mixed sex). Cages were held in I&Q rearing rooms at 25 °C, 55% RH, and a 16:8 h (L: D) photoperiod for approximately 2 weeks until sufficient ACP adults were produced to use in experiments.

### Insect Irradiation

All movement of ACP to facilitate the irradiation process was conducted under a California State Plant Pest Movement Permit #4033. Mixed-sex cohorts of 1- to 2-day-old adult ACP were aspirated into 40-dram amber pharmaceutical vials (1.80 cm internal diameter × 4.0 cm height; United States Plastic Corporation, Lima, OH). The temporal emergence pattern of male and female ACP adults is very similar, fostering the collection of approximately equal numbers of males and females, and at 2 days old, the majority of adults have not yet reached sexual maturity (Wenninger and Hall 2007). For the laboratory-cage assays, each vial housed 50 individuals, versus approximately 800 per vial for the field-cage assays (assuming 5% to 6% mortality, the goal for these field-cages was to release 750 sterile individuals from each vial; Supplementary Tables S1 and S2). Vials were sealed with child-resistant closure (CRC) caps, and humidity in the vials was maintained by lining the inner surface of each cap with moistened filter paper (70 mm diameter). The caps were further secured using transparent adhesive tape and each vial was marked with its specified radiation dose. The vials were subsequently stacked in a cardboard box, enclosed within a large reclosable plastic bag, and placed into an electric (12 V DC), portable cooler maintained at 6.5 °C. A data logger was included inside the cooler to monitor temperature and humidity. The psyllids were then transported by car to the USDA-APHIS-PPQ facility in Salinas, CA, for irradiation the following day. Irradiation was performed using a Radsource 2400 X-ray irradiator (Radsource Technologies, Buford, GA, USA), delivering a dose rate of 14.76 Gy min^−1^, as detailed in Ferrater et al. (2024). Across the experiments, 3 target doses were administered; 240 and 320 Gy for both laboratory and field-cage experiments, and 400 Gy for the laboratory cage experiments only. These radiation doses were selected based on previous work on ACP radiation biology (Ferrater et al. 2024). Post-irradiation, the psyllids were transported back by car to UCR. The vials were subsequently allowed to acclimate to room temperature for at least an hour before the irradiated ACP were released into the experimental cages. Total time from initial collection to release was approximately 40 to 48 h, and from irradiation to releases 20 to 24 h.

### Laboratory Cage Experiments

The impact of a single release of sterile ACP at different OFRs on the growth of “wild” ACP populations was initially examined in small laboratory populations in insect rearing cages measuring 47.5 × 47.5 × 47.5 cm each containing fifteen 6-month-old *B. koenigii* seedlings in an active growing stage with young foilage suitable for ACP reproduction. Five female and 5 male, 2- to 3-day-old adult ACP, were collected from the UCR colony (initiated in 2011), and introduced into each cage. These non-irradiated ACP represented an adventive “wild” population. At the same time, mixed-sex batches of irradiated adults (∼1:1 sex ratio), exposed to 240, 320, or 400 Gy, were introduced at OFRs of 5:1, 10:1, and 15:1 (sterile: fertile); resulting in the release of 50, 100, and 150 sterile individuals per cage, respectively. This resulted in 9 combinatorial (dose × OFR) treatments (Supplementary Table S1). A control treatment comprising of only the 10 non-irradiated adults was also included as a baseline against which to compare subsequent population growth. The treatments were arranged randomly with 3 replicates of each, and the entire experiment was repeated on 2 occasions (in April 2023 and June 2024). Following the introduction of the psyllids, the cages were maintained in a growth room at 25 °C, 55% RH, and a 16:8 h (L: D) photoperiod. ACP were allowed to mate and oviposit for 7 days undisturbed. Attempts were then made to remove all parental adults from each cage, using an aspirator to collect individuals directly from the plants, or from the walls of the cage after gently tapping the plants to encourage the adults to fly. During this process care was taken to not displace any juvenile life stages (eggs and nymphs) and while it is possible that the occasional cryptic parent escaped removal, the number was minimized by a second check, a further 3 days later. Cages were returned to the growth room and the immature psyllids contained within were monitored and allowed to mature. In the first round of the experiment (April 2023), the size of the F1 population in each cage was estimated 32 days post introduction of the parents, based on known ACP egg-to-adult development times (Liu and Tsai 2000, Hall and Abrigo 2007). All adult ACP within each cage were aspirated into a collecting vial, euthanized at −20 °C, and subsequently counted under a stereomicroscope. In the second round of the experiment (June 2024), the size of the F1 adult population in each cage was estimated in the same manner, 35 days post introduction of the parents.

### Field Cage Experiments

To better replicate the challenges that SIT individuals may face if they were released in the field, we scaled up our laboratory study, releasing more psyllids, onto full size mature lemon trees, enclosed within secure field cages (Fig. 1). At the outset, 20 cages were each infested with 150 non-irradiated, newly-emerged mixed-sex adult ACP (again representing an adventive wild population) and an appropriate number of mixed-sex irradiated adults (determined by overflooding ratio) on a single release event. The irradiated psyllids were exposed to either 240 or 320 Gy of X-ray radiation, and were introduced at OFRs of either 10:1 or 15:1 (based on findings from the laboratory cages), corresponding to the release of 1,500 or 2,250 sterile individuals per cage, respectively. This resulted in 4 combinatorial (dose × OFR) treatments, and a control treatment comprising of only the 150 non-irradiated adults was again included as a baseline against which to compare subsequent population growth (Supplementary Table S2). Treatments were arranged in a randomized complete block design, with 3 replicates of each initiated in Spring 2024, and 4 replicates initiated in Summer 2024 (Supplementary Table S2). Following the release of the parental ACP, degree-day accumulations were generated using a degree-day model to predict the ongoing phenological development of the caged populations (UC IPM 2025a). This procedure entails summing daily heat units to estimate the timing of egg-hatch, nymphal development, and adult emergence, over subsequent generations using lower and upper ACP developmental thresholds of 11.1 °C and 32.2 °C (52°F and 90°F, respectively; Tsai et al. 2000). Meteorological inputs were obtained from the nearest California Department of Water Resources weather station (U.C. Riverside, CIMIS #44), degree-days were calculated with the single-sine (horizontal cutoff) method (UC IPM 2025b) and used to predict the population peak for the F1 generation. This peak was targeted for estimating the size of each caged ACP population.

Sampling followed the protocol of Milosavljević et al. (2018) with slight modifications. Since the immature ACP lifestages are not easily displaced from a plant, eggs and nymphs were assessed via destructive sampling. Each tree canopy was conceptually divided into 4 quadrants (north, south, east, and west), and 5 flush shoots were randomly collected from each quadrant, yielding 20 shoots per tree. The excised shoots were then immediately isolated in individual reclosable polyethylene bags, and stored at 4 °C (halting ACP development). The flushing stage of each excised shoot was later assessed and scored according to the scale (1 to 5) proposed by Cifuentes-Arenas et al. (2018), where 5 represent the most mature shoot stage. Each shoot was then carefully examined under a stereomicroscope and the numbers of unhatched eggs, early instar nymphs (1st—3rd), and late-instar nymphs (4th—5th) attached to the shoots, were recorded. In contrast, adult density in the field cages was estimated from the numbers of ACP caught on yellow panel traps positioned on the 4 inner walls of the cages. Traps were installed at a height of around 1.5 m and left in place for 7 days.

### Data Analysis

All analyses were conducted in the R programming language, version 4.5.0 (R Core Team 2025).

#### Laboratory Cages

Two rounds of experiments were conducted, each with 10 combinatorial treatments (dose × OFR; Supplementary Table S1). In both years, F1 adult abundance was estimated at approximately 1 month after initially releasing ACP into the cages (32 d in 2023, 35 d in 2024). F1 adult counts in the 2 experiments were analyzed using a generalized linear model with a fixed effect of treatment, year as a blocking effect, and negative binomial error to appropriately capture the count nature of the data (Crawley 2007; glm.nb()). The full model was compared to a series of reduced models, missing one of the variables, using likelihood- ratio tests to identify the minimum adequate model (Crawley 2007). A significant effect was followed up with pairwise pre-planned contrasts between the non-irradiated control and each treatment combination evaluated at *α* = 0.05.

#### Field Cages

The impact of a single SIT release on ACP population densities on caged citrus trees was investigated in experiments conducted in spring 2024, and again in summer 2024. The spring releases consisted of 3 replicate cages for each of 5 combinations of irradiation dose and OFR (dose × OFR; Supplementary Table S2), while the summer releases had 4 replicates of each treatment. Counts of ACP adults on traps were analyzed at the cage level using a generalized linear model (GLM) with fixed effects of treatment (ie radiation by overflooding ratio combination), season (ie spring or summer experiment), a treatment by season interaction, and negative binomial error (Crawley 2007). Likelihood-ratio tests were used to compare the full model to a series of reduced models to identify the most parsimonious adequate description of the data. A significant effect of treatment or treatment by season interaction was followed up with a comparison of the estimated marginal means [emmeans()] of each treatment combination relative to the control group within a given generation.

Counts of eggs, early-instar nymphs (1st—3rd instars), and late-instar nymphs (4th and 5th instars) on 20 shoots per tree collected from each cardinal direction were analyzed using separate generalized linear mixed-effects models [GLMM; glmmTMB()], which included fixed effects of treatment, season, a treatment by season interaction, and flush age category (1 through 5, with lower values equating to younger tissue) on the sampled shoot as a covariate (Pinheiro and Bates 2000). A random effect of quadrant (ie cardinal direction) nested within cage was included to capture autocorrelation stemming from repeated subsampling (ie 20 subsamples) from the same trees, and negative binomial error. Again, model simplification was used, and significant main effects were followed up with pairwise comparison of the estimated marginal means of each treatment combination relative to the control within a given generation.

## Results

### Laboratory Cages

**Fig. 2.**
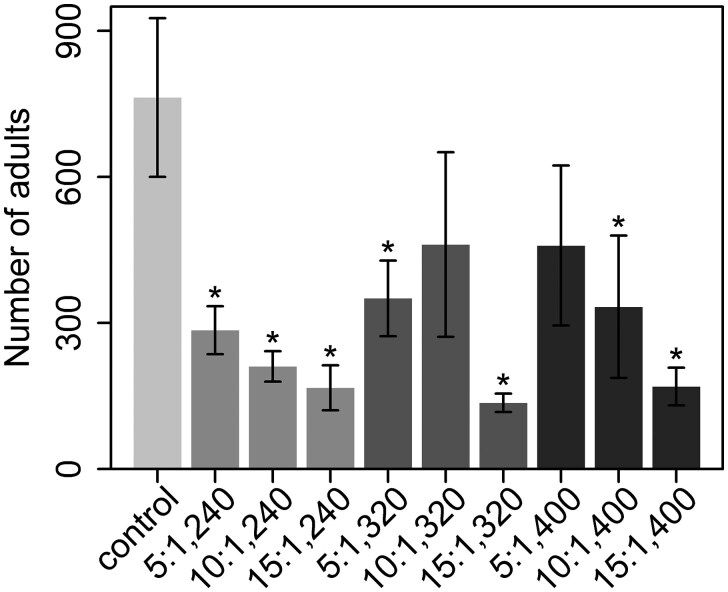
Effect of radiation dose and release rate (OFR) on the mean (±SE) number of F1 adult ACP per cage based on a generalized linear model with negative binomial error. Treatments denoted with an * differed significantly from the non-irradiated control based on pre-planned paired contrasts evaluated at *α* = 0.05.

The impact of releasing sterile ACP on the growth of a wild ACP population was assessed by direct quantification of the resulting F1 adult generation. The blocking effect of year was not significant and was dropped during model simplification, with the final model including only a significant effect of treatment on the number of F1 adults (*χ*^2^ = 33.331, *df* = 9, *P* < 0.0001). Across the 2 experimental repetitions (2023 and 2024), 7 of the 9 treatments (dose × OFR combinations) resulted in significantly lower numbers of F1 adults compared to the control (Fig. 2). Overall, OFR appeared to have at least as strong of an effect as dose, with higher OFR always trending toward lower F1 population counts (Fig. 2). The combination of 320 Gy and 15:1 OFR resulted in the greatest observed reduction compared to the control with approximately 82% fewer F1 adults (Fig. 2).

### Field Cages

Across treatments and seasons, ACP juvenile counts ranged between 0 and 465 eggs per shoot (mean ± SD: 15.21 ± 42.44), between 0 and 375 early-instar nymphs (17.47 ± 43.53), and between 0 and 197 late-instar nymphs (4.80 ± 17.51). Overall, the mean (±SD) flush category was 2.6 (±1.05), with slightly younger flush during the spring (2.4 ± 1.14) compared to the summer (2.75 ± 0.96). Adult counts ranged between 0 and 166 among traps (38.83 ± 51.75). The final models for egg counts, early-instar nymphs, and late-instar nymphs all included significant effects of treatment (dose × OFR), season, and flush stage (Table 1). The treatment-by-season interaction was dropped for each model during model simplification. For adults, the final model included significant effects of treatment, season, and a treatment-by-season interaction (Table 1).

**Table 1.** Test statistics and significance values for separate generalized linear models with negative binomial error describing the effects of an SIT release on the abundance of different ACP life stages in spring and summer 2024 field-cage experiments

	Eggs			1st-3rd instars			4th-5th instars			Adults		
Source	*χ*2	*df*	*P*	*χ*2	*df*	*P*	*χ*2	*df*	*P*	*χ*2	*df*	*P*
**Treatment**	38.864	4	<0.0001	60.161	4	<0.0001	57.547	4	<0.0001	31.889	4	<0.0001
**Season**	201.54	1	<0.0001	301.04	1	<0.0001	137.40	1	<0.0001	78.829	1	<0.0001
**Flush**	45.812	1	<0.0001	43.105	1	<0.0001	6.662	1	0.0098	…^a^	…	…
**Trt × Seasn**	…^b^	…	…	…^b^	…	…	…^b^	…	…	11.654	4	0.0201

a Term not evaluated.

b Term dropped during model simplification.

**Fig. 3.**
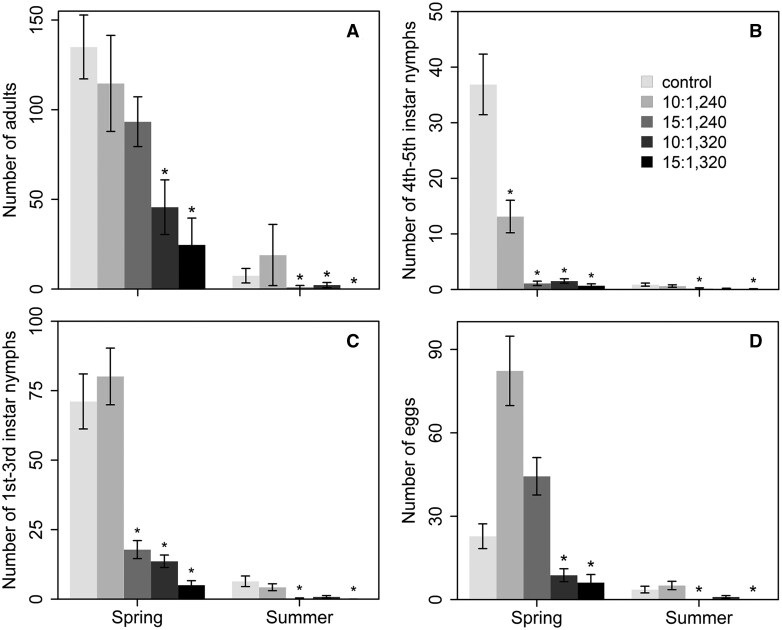
Effects of treatment combination (overflooding ratio, irradiation dose) and season, in separate generalized linear models with negative binomial error, on mean (±SE) number of ACP A) adults per trap, B) late-instar nymphs per shoot, C) early-instar nymphs per shoot, and D) eggs per shoot in the spring and summer 2024 field-cage releases. Note the scale of the y-axis differs among panels. “*” denotes significant differences in counts between treatment groups and untreated controls within a given season based on pairwise comparisons of estimated marginal means with Bonferroni correction to adjust for multiple comparisons at *α*_c_ = 0.0125.

**Fig. 4.**
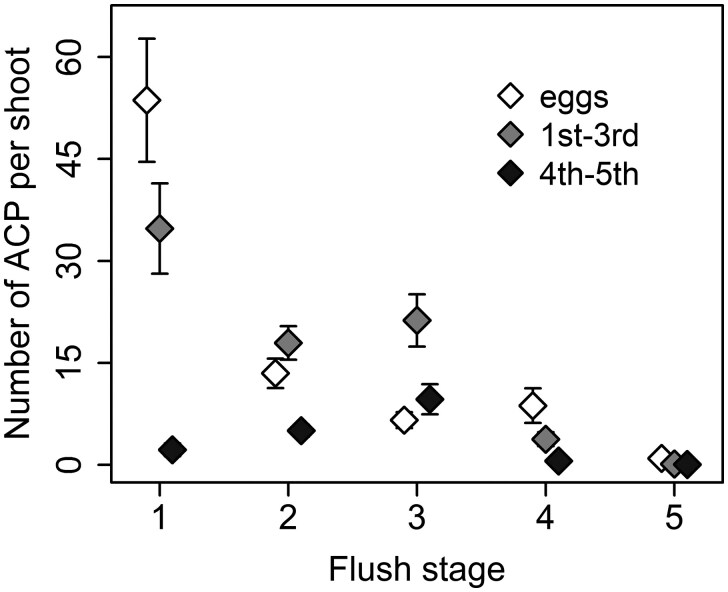
Effects of citrus flush stage (higher values equate to more mature foliage) on mean (±SE) counts of ACP eggs, early-instar nymphs, or late-instar nymphs across the spring and summer releases in separate generalized linear models with negative binomial error. Points offset slightly for clarity.

Across all treatments, counts of all ACP life stages were consistently higher in the spring compared to the summer, nearly 17-fold overall for eggs, 16-fold for early-instar nymphs, more than 27-fold for late-instar nymphs, and nearly 14-fold for adults (Fig. 3). Among radiation dose-overflooding ratio treatments, adult counts in both 320 Gy treatments were significantly lower than the control in spring and 3 of the SIT treatments resulted in significantly lower counts in the summer (Fig. 3A). All 4 SIT treatments resulted in significantly lower late-instar nymphal counts compared to the control (Fig. 3B), compared to 3 of the treatment combinations for early-instar nymphs (Fig. 3C), and both of the 320 Gy treatments for eggs (Fig. 3D). Among the treatments, the combination of 320 Gy and 15:1 OFR reduced ACP counts the most compared to the control, with an approximate 80% reduction for eggs, more than 90% for early-instar nymphs, approximate 98% for late-instar nymphs, and more than 80% for adults (Fig. 3). Across all juvenile life stages, ACP abundance was negatively associated with the flush stage of the sampled shoots (Fig. 4), but the effects were more pronounced for eggs (coefficient ± SE: −0.447 ± 0.066) and early-instar nymphs (−0.341 ± 0.052) than for late-instars nymphs (−0.206 ± 0.079).

## Discussion

Previous work has identified that male and female ACP can effectively be rendered sterile, using an appropriate dose of X-ray radiation (320 Gy), without significantly impacting their ability to mate, demonstrating the potential for developing SIT as a strategy for managing invasive populations of this pest (Ferrater et al. 2024). The present study builds on that foundational work and confirms that the growth of caged ACP populations, in both laboratory and field-cage settings, can be suppressed if sterilized individuals are released in sufficient numbers. We tested the ability of a single release of sterile ACP at various radiation doses and overflooding ratios (OFRs) to control surrogate adventive wild populations. Initial small-cage experiments with curry leaf seedlings showed that in 7 of 9 treatment combinations (dose × OFR), the release of sterile psyllids significantly reduced F1 populations, with psyllids exposed to 320 Gy and released at 15:1 OFR achieving 82% suppression versus controls. Subsequent scaled-up trials, conducted in larger field cages containing mature citrus trees and greater numbers of wild ACP, replicated these results, with 320 Gy and 15:1 OFR again proving most effective.

Overall, the significant suppressive effect that releasing sterilized ACP had on the growth of wild ACP populations, in both laboratory and field-cage experiments, was encouraging and supports the continued development of SIT as a tool for controlling ACP. Our laboratory experiments demonstrated that, given an adequate dose of radiation to induce sterility, increasing the OFR resulted in fewer F1 adults. In those experiments, since most, if not all, parental ACP were removed from the laboratory cages, we can be almost certain that only the F1 generation of ACP was counted, and as an initial foray, our findings were consistent with the basic expectation of SIT, that a higher OFR increases the probability of wild × sterile mating (Hendrichs et al. 2002, Carpenter et al. 2005, Robinson 2021). Furthermore, the finding suggests that, at least when produced/handled according to our protocols and assayed in a tightly controlled environment, ACP subjected to 320 Gy of X-ray radiation remained healthy enough following their release, to compete with their wild counterparts for mating opportunities. For SIT to be a success, a careful balance must be struck between maximizing individual sterility and minimizing the effects of irradiation on insect competitiveness. In addition to triggering mutations in an organism’s DNA, radiation exposure also impacts other cellular processes like metabolism, that in turn may affect an individual’s ability to survive in the wild, to navigate its natural environment in order to find mates, and having located a mate, to engage in pre- and post-copulatory competition with wild counterparts (Chen et al. 2023, Mainardi et al. 2023, Peccerillo et al. 2023). In addition, the mass-rearing process and handling of the insects during the sterilization process could further compromise insect quality and mating competitiveness (Calkins et al. 2005, FAO/IAEA/USDA 2019).

Our field-cage experiments again demonstrated that, given an adequate dose of radiation to induce sterility, increasing the OFR resulted in fewer F1 individuals. However, they also demonstrated that estimating the impact of an SIT release under variable field conditions is more complicated. Timing of SIT releases (spring or summer) and the choice of ACP life stage to census, clearly impacted the apparent extent of any suppressive effect. In simple terms, this was exemplified by multi-fold differences in the counts of each life stage in the field-cage experiments (particularly the control cages) initiated in summer versus those initiated in spring. This was not entirely surprising and reflects typical declines in wild ACP populations in Southern California during the summer months when psyllids are generally less abundant and less reproductively active, in response to lower availability of young citrus flush (Milosavljevic et al. 2018). However, this highlights the importance of young citrus flush for ACP reproduction and development, and the impact this has on the overall abundance and spatial distribution of ACP. Furthermore, flush-induced effects in summer are likely to be further exacerbated by increased mortality rates across the life stages due to extreme heat. Daytime temperatures at the study site in Riverside regularly exceed 40 °C (104°F) during the summer months in 2024. Mortality rates approaching 100% have been reported for ACP in Florida laboratory colonies kept at 43 °C (109°F) for 3 h, although the extent to which this finding applies to field populations is unknown (Hall and Hentz 2014). Certainly, a portion of the population does survive each year. During hot periods and in the absence of flush, adult ACP likely seek the shelter of inner branches, mate less, lay fewer eggs, and perhaps survive longer (Weninger and Hall 2007, Hall et al. 2011, Antolinez et al. 2021). This emphasizes that the timing of sterile insect releases relative to host plant phenology is likely to significantly influence the outcome of SIT. For an SIT program against ACP to be effective, releases of sterile insects must clearly be tied to the timing of the citrus flush. For most commercial citrus, the timing of flushing is predictable, although it may differ across varieties (de Carvalho et al. 2020), but flushing may be less predictable in urban environments where tree maintenance (eg pruning, watering, and the application of fertilizer) is expected to be less coordinated.

A further complicating factor in the field-cage experiments was the almost certain presence of overlapping adult generations. While it is fair to assume that all eggs and nymphs in our F1 census counts were indeed F1 offspring, it is likely that the adult counts included a portion of surviving parental ACP. Control cages were seeded with just 150 wild ACP, but our treatment cages received the same number of wild ACP plus 10× or 15× as many sterile psyllids. Although not contributing to the production of the F1 generation, any surviving sterile adults are open to misidentification as belonging to the F1 generation because we had no way of distinguishing them from genuine F1 adults. Thus, adult counts likely under-estimate the impact of SIT. Estimates relying on egg counts may also be unreliable since we know that wild females that have mated with a sterile male will continue to lay a similar number of eggs, albeit non-viable ones (Ferrater et al. 2024). Therefore, only the number of viable eggs is useful, but we were unable to make that distinction in our counts. The life stage that is likely to provide the most reliable estimate of the F1 population in our cages is late-instar nymphs. Since we only introduced adults to the cages, any late-instar nymphs at the census point are true F1 offspring of a suitable genetic constitution to have avoided early death (ie at the egg or early-instar stage) and we would expect the vast majority to progress to adulthood. Looking to the future, this finding has important implications for monitoring the efficacy of potential SIT releases in the field, suggesting that relying on a single life stage may under- or overestimate suppression. Future studies may consider marking the released adult ACP in such a way that they can later be identified (Nakata 2008, Hagler et al. 2024).

Although suppression of the F1 generation following a single release of sterile ACP was strong and consistent across treatments in both laboratory- and field-cage experiments, it is important to note from the latter that no combination of dose and OFR resulted in complete eradication. ACP are polyandrous (Wenninger and Hall 2008) and any wild female that mates with a wild male, even if she subsequently mates with a sterile male, is likely to produce some viable offspring. Without further releases of sterile individuals, these viable offspring are subsequently likely to mate with each other leading to a rebound in the wild population. Indeed, during the process of cleaning up the field cages several months after the experiments were completed, it became evident that active ACP were still present. In an operational program, such residual fertility in the population could be countered by repeated sterile insect releases, perhaps on a weekly basis, again targeting peak flushing periods (eg from March through April). This tactic of multiple releases aligns with established protocols for other pest systems managed with SIT (eg tephritid fruit flies, tsetse flies, screwworm, and moths) to counteract population rebound and immigration (Klassen and Curtis 2005, Vreysen et al. 2007). A further difference between our experiments and an operational SIT program is that in all our cages, we had an exact count of the number of wild ACP that we were seeking to combat. Thus, our ORFs were very precise. In the open environment, following initial detection of wild ACP in an area (eg via state-deployed yellow panel traps), the numbers of wild ACP in the target area would need to be estimated using counts collected using visual survey methods. The accuracy of such estimates will have implications for the numbers of SIT individuals released and, as a result, potentially the success of those releases. Again, serial SIT releases targeting peak flushing periods may help to ensure success.

This study provides compelling evidence of the potential SIT holds for the suppression of ACP populations, albeit under controlled, closed conditions that may not fully replicate the complexities of open-field environments, such as immigration, emigration, and other environmental factors. With the right balance of radiation dose, release ratio, and timing of releases relative to citrus flush periods for optimal effectiveness, SIT could offer a viable IPM tool for managing ACP in urban buffer zones, and potentially also eradicating the pest in newly infested areas. Further refinement of SIT methodology, handling, and storage protocols for sterile ACP is necessary to enhance its practicality and sustainability as a component of ACP control strategies. One challenge will be to provide robust evidence that irradiated psyllids will not contribute to spreading HLB after they are released. Currently, we utilize only ACP which have been reared to adulthood on *B. koenigii* prior to sterilization and release. This is done to ensure that at the point of release, all SIT individuals are free from *C*Las, and because previous work has shown that, if normal (ie non-irradiated) ACP acquire *C*Las as adults, they are highly unlikely to transmit the pathogen to new hosts within 28 days (Ammar et al. 2016), a period beyond the expected survival time of SIT individuals (Ferrater et al. 2024). However, it is also uncertain how the X-ray radiation required to produce sterile ACP affects their capacity to transmit *C*Las. In another psyllid species, *Bactericera cockerelli*, that also vectors a *Ca*. Liberibacter pathogen to plants, radiation exposure was recently shown to significantly reduce vector competence (Chang et al. 2023). No mechanism was identified but radiation-induced changes to the physiology, immune response, and microbiome of the psyllids, could all potentially alter acquisition, development, and survival of the pathogen (Cooper et al. 2023, Kwak et al. 2025). Empirical data to support a similar finding in ACP would be extremely valuable.

Another challenge will be to improve the mass-rearing process to meet the demands of an operational program. While the size of an ACP mass-rearing rearing program needed for an operational control program is unknown at this time, it will depend on factors such as the size of the wild population in a given area, the size of the treated area, and the strategic purpose of using an SIT tool against ACP which will all influence the size, scope, and potential costs of a future program (Hendrichs et al. 2021). Currently, ACP are still mass-reared on plants. This worked fine for our study and could likely also support small scale operational releases, but it contrasts with established SIT programs where insects are typically reared on artificial diets. Artificial diets have been developed that can support different ACP development stages at least for short periods (Pelz-Stelinski et al. 2010), but achieving complete development from nymph to adult remains elusive (Hall et al. 2010, Russell and Pelz-Stelinski 2015). Without an artificial diet, advancement of an SIT program for ACP will require significant investment in greenhouse space to house sufficient numbers of host plants (eg curry leaf). An alternative may be to mass-rear psyllids inside field-cages similar to those used for our experiments, where up to 100,000 ACP can be reared per caged tree (R. Pandey, personal communication). That said, having an artificial diet and/or the capacity to rear millions should not be seen as a strict requirement to mount an effective control program as there are examples of successful smaller scale operational programs where smaller numbers of sterile insects were released to achieve a strategic goal as part of an area-wide control program (Suckling et al. 2007). With investment in the right areas (in both research and infrastructure), we believe that SIT can contribute significantly to managing ACP in southern California and preventing the establishment of HLB in commercial citrus production areas.

## Supplementary Material

toag116_Supplementary_Data
