# Supplementary material for: Development of the sterile insect technique as a control strategy for the Asian citrus psyllid: determining the number of sterile individuals needed to suppress the growth of wild populations
Source: J Econ Entomol. 2026 Apr 27;119(4):2524–34. doi: 10.1093/jee/toag116 (PMC13462264; doi:10.1093/jee/toag116)
Supplement: Supplementary file 1 [file toag116_supplementary_data.docx]

**Supplementary Material**

**Table S1.** Three radiation doses and three overflooding ratios (OFR) were combined to evaluate suppression of a wild ACP population inside small laboratory cages harboring *B. koenigii* plants.

| **Treatment (Dose, OFR)** | **Wild ACP** | **Irradiated ACP (per cage)** | **Replicates (2023)** | **Replicates (2024)** |
| --- | --- | --- | --- | --- |
| Control (0 Gy, 0:1) | 10 | - | 3 | 3 |
| 240 Gy, 5:1 | 10 | 50 | 3 | 3 |
| 240 Gy, 10:1 | 10 | 100 | 3 | 3 |
| 240 Gy, 15:1 | 10 | 150 | 3 | 3 |
| 320 Gy, 5:1 | 10 | 50 | 3 | 3 |
| 320 Gy, 10:1 | 10 | 100 | 3 | 3 |
| 320 Gy, 15:1 | 10 | 150 | 3 | 3 |
| 400 Gy, 5:1 | 10 | 50 | 3 | 3 |
| 400 Gy, 10:1 | 10 | 100 | 3 | 3 |
| 400 Gy, 15:1 | 10 | 150 | 3 | 3 |

**Table S2.** Two radiation doses and two overflooding ratios (OFR) were combined to evaluate the suppression of a wild ACP population inside field cages harboring a mature lemon tree.

| **Treatment**  **(Dose, OFR)** | **No. of wild ACP** | **No. of irradiated ACP** | **No. of cages (spring/summer)** |
| --- | --- | --- | --- |
| Control (0 Gy, 0:1) | 150 | - | 3 / 4 |
| 240 Gy, 10:1 | 150 | 1500 | 3 / 4 |
| 240 Gy, 15:1 | 150 | 2250 | 3 / 4 |
| 320 Gy, 10:1 | 150 | 1500 | 3 / 4 |
| 320 Gy, 15:1 | 150 | 2250 | 3 / 4 |
